# Supplementary material for: Adaptation and Validation of a French Version of the Vaccination Attitudes Examination (VAX) Scale
Source: Vaccines (Basel). 2023 May 19;11(5):1001. doi: 10.3390/vaccines11051001 (PMC10221281; doi:10.3390/vaccines11051001)
Supplement: Supplementary file 1 [file vaccines-11-01001-s001.zip › vaccines-2381760-supplementary.pdf]

Supplementary Data

Table. Descriptive characteristic of the VAX total score by gender and age categories for the total sample (n= 450).

	Vax total score
	Means ± SD [min – max]
Sexe	
Female	28.6±10.3 [13-58]
Man	32.6±11.3[12-60]
Age, years old	
18-24	30.78±10.45 [14-56]
25-34	29.3±9.65 [12-59]
35-44	31.77±11.6 [13-56]
45-54	31.85±13.39 [15-60]
55-64	33.08±14.07 [15-60]
65-74	32.72±14.43 [19-60]
75-81	27.25±9.33 [19-49]

Note. Data are presented as mean± standard deviations [range]; VAX = Vaccination Attitudes Examination scale ;
